# Notes on the leaf insects of the genus *Phyllium* of Sumatra and Java, Indonesia, including the description of two new species with purple coxae (Phasmatodea, Phylliidae)

**DOI:** 10.3897/zookeys.913.49044

**Published:** 2020-02-19

**Authors:** Royce T. Cumming, Sarah Bank, Stephane Le Tirant, Sven Bradler

**Affiliations:** 1 Associate Researcher, Montréal Insectarium, 4581 rue Sherbrooke est, Montréal, Québec, Canada, H1X 2B2; 2 Richard Gilder Graduate School, American Museum of Natural History, New York, NY 10024, USA; 3 Department of Animal Evolution and Biodiversity, Johann-Friedrich-Blumenbach Institute for Zoology and Anthropology, University of Göttingen, Untere Karspüle 2, 37073 Göttingen, Germany; 4 Collection manager, Montréal Insectarium, 4581 rue Sherbrooke, Montréal, Québec, H1X 2B2, Canada

**Keywords:** Cryptic species, molecular phylogeny, phasmid, taxonomy, walking leaf

## Abstract

Within the last two years, the leaf insects of the genus *Phyllium* of both the islands of Java and Sumatra have been reviewed extensively based on morphological observations. However, cryptic species which cannot be differentiated morphologically may be present among the various populations. Since it has frequently been demonstrated that analyses based on molecular data can bring clarity in such cases, we conducted a phylogenetic analysis based on three genes (nuclear gene 28S and mitochondrial genes COI and 16S) from the *Phyllium* species of these islands. The results show distinct molecular divergence for several populations and suggest the presence of two new cryptic species, morphologically inseparable from *Phylliumhausleithneri* Brock, 1999. From Sumatra, the population originally thought to be a range expansion for *Phylliumhausleithneri*, is now here described as *Phylliumnisus***sp. nov.**, with the only consistent morphological difference being the color of the eggs between the two populations (dark brown in *P.hausleithneri* and tan in *P.nisus***sp. nov.**). Further, an additional population with purple coxae from Java was morphologically examined and found to have no consistent features to separate it morphologically from the other purple coxae species. This cryptic species from Java was however shown to be molecularly distinct from the other purple coxae populations from Sumatra and Peninsular Malaysia and is here described as *Phylliumgardabagusi***sp. nov.** In addition, *Phylliumgiganteum* is here officially reported from Java for the first time based on both historic and modern records of male specimens.

## Introduction

**Figure 1.**
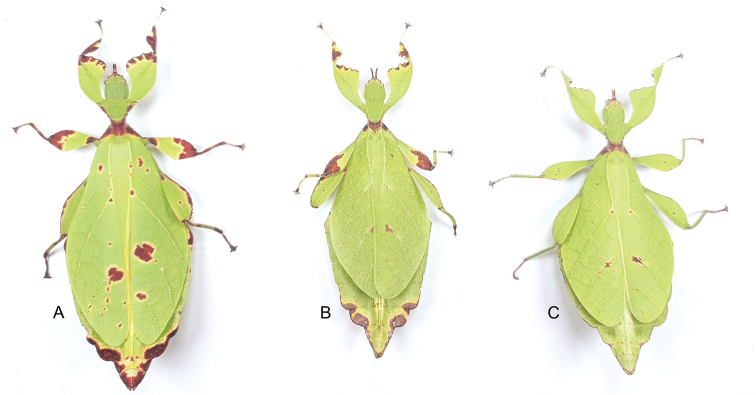
Three female *Phyllium* with purple coxae, dorsal. Bred by Tim Van molle (Rupelmonde, Belgium). **A***Phylliumhausleithneri* “Tapah, Perak Malaysia” **B***Phylliumnisus* sp. nov. “Bukit Daun, Sumatra, Indonesia” **C***Phylliumgardabagusi* sp. nov. “Argopuro, Java, Indonesia”.

Stick and leaf insects (Phasmatodea) are known for their extreme forms of crypsis by camouflaging themselves as parts of plants, with the majority of forms imitating twigs and exhibiting extremely slender and elongated bodies (Bedford 1978). In contrast, the true leaf insects (Phylliidae) are excellent leaf mimics with extensive lobe-like expansions throughout their bodies and legs, forming a dorso-ventrally flattened body (Figure 1). Leaf similarity is further enhanced by the wing venation of the large female forewings resembling the nerves of angiosperm leaves (Klante 1976). Historically, Phylliidae have been considered to be an early lineage among Phasmatodea branching off from a basal node of the phasmatodean tree of life (Zompro 2004). However, more recent phylogenomic analyses place them as a subordinate lineage among the Euphasmatodea as members of the Old World clade Oriophasmata (Simon et al. 2019). The geographic distribution of the currently described 80+ extant species of Phylliidae (Brock et al. 2019) ranges from as far east as the Seychelles in the Indian Ocean, throughout the Australasian region, to as far west as Fiji in the Southern Pacific (Günther 1953) with extinct members occurring in the Eocene of Germany (Wedmann et al. 2007).

Previous studies have dealt with the Phylliidae of Indonesia in several regional works in the last few years [Wallacea: Cumming et al. 2019; Java: Cumming and Le Tirant 2018; Sumatra: Cumming et al. 2018 and Seow-Choen 2018; Lesser Sunda Islands: Cumming et al. 2018; and West Papua: Brock 2014; and van de Kamp and Hennemann 2014], however, only morphological characters have been reviewed to date. This work reviews the *Phyllium* species of the Indonesian islands of Sumatra and Java using molecular data and identifies two cryptic species which were morphologically inseparable from *Phylliumhausleithneri* in need of formal description.

## Materials and methods

### Morphological examination

Morphological examinations were done with a Leica ZOOM 2000 stereomicroscope. Measurements of the holotype were conducted to the nearest 0.1 mm using digital calipers. Egg orientation terminology follows Clark (1978). Wing venation terminology follows that of Burt (1932) and Ragge (1955). Type material coloration descriptions are based on photographs of live individuals bred in captivity by Bruno Kneubühler (Switzerland), Tim Van molle (Belgium), and from well preserved dried specimens.

### Abbreviations

The following institutional abbreviations are used herein:

**IMQC** Insectarium de Montréal, Montréal, Québec, Canada

**MZSF** Strasbourg Zoological Museum, Strasbourg, France

**NHMB** Naturhistorisches Museum Basel, Basel, Switzerland

**Coll RC** Private collection of Royce T. Cumming, U.S.A.

**Coll SLT** Private collection of Stéphane Le Tirant, Canada

The following wing venation abbreviations are used in Figure 10 (listed in order from the anterior to the posterior of the wing):

**C** costa

**Sc** subcosta

**R** radius

**R1** first radius

**Rs** radial sector

**M** media

**MA** media anterior

**MP** media posterior

**Cu** cubitus

**CuA** cubitus anterior

**CuP** cubitus posterior

**Cu+1AA** cubitus fused with first anterior anal)

**1AA–7AA** anterior anal veins one through seven

**1PA–5PA** posterior anal veins one through five

**1A** first anal

### Molecular laboratory work and phylogenetic analysis

We selected 18 *Phyllium* specimens that represent all eight species known from Java and Sumatra (see species checklist at the end for more details and Suppl. material 1: Table S1 for specimen data). Outgroups were chosen according to Simon et al. (2019), who recovered Bacillinae as sister taxon to Phylliidae, whereas Anisacanthinae is sister to Bacillinae + Phylliidae, and Aschiphasmatinae forms the sister group to all remaining Euphasmatodea.

One dried hind leg per specimen was soaked in water before removing femoral muscle tissue. Genomic DNA was extracted from the muscle tissue with the Quick-DNA^TM^ Miniprep Plus kit (Zymo Research, Irvine, USA) and eluted in 60 µl elution buffer following the manufacturer's protocol. Using PCR, we amplified the two mitochondrial genes COI and 16S and parts of the nuclear gene 28S using primers described elsewhere (see Suppl. material 2: Table S2). After quality assessment via gel electrophoresis, positive PCR products were purified with Exo-SAP-IT^TM^ (Thermo Fisher Scientific, Waltham, USA) and subsequently Sanger-sequenced (Microsynth Seqlab, Göttingen, Germany). DNA sequences were deposited in GenBank under accession numbers MN364958–MN365012 (Suppl. material 1: Table S1).

Sequences were aligned for each individual gene with ClustalW v. 2.1 (Larkin et al. 2007) implemented in Geneious v. 11.0.5 (http://www.geneious.com). The alignments were trimmed using a custom Perl script and concatenated using FASconCAT (Kück and Meusemann 2010). The phylogenetic tree was inferred in IQ-TREE v. 1.6.8 (Nguyen et al. 2015) using the suggested substitution model GTR+F+G4 (Kalyaanamoorthy et al. 2017) and the ultrafast bootstrap approximation for branch support (Hoang et al. 2017).

## Results

### Phylogenetic analysis

**Figure 2.**
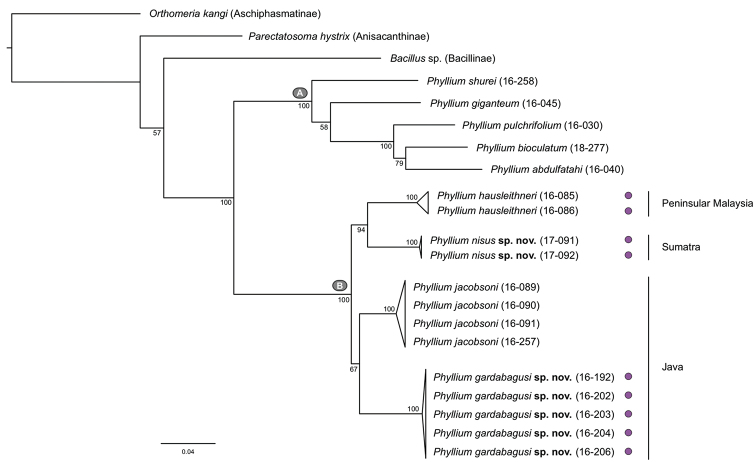
Maximum likelihood tree of 18 *Phyllium* specimens from Java and Sumatra and three outgroup species. The phylogenetic tree was generated with IQ-TREE and rooted with *Orthomeriakangi* (Aschiphasmatinae). Ultrafast Bootstrap support values are given below nodes. Lineages with short branch lengths were collapsed. **A** and **B** depict the two main *Phyllium* clades. Purple circles indicate those species with purple colored coxae.

For the 21 specimens sampled (18 *Phyllium* and three outgroups), we obtained 16 COI, 18 28S, and 21 16S sequences, resulting in a final concatenated dataset comprising 1758 bp (Suppl. material 5 for the supermatrix; Suppl. material 6 for the original tree file). The phylogenetic tree recovers Phylliidae as a monophyletic group with excellent support (Figure 2). Within Phylliidae, some node support values are suboptimal but can be neglected, since our primary objective was to reveal specific species clusters rather than phylogenetic relatedness. Clusters with short branch lengths were collapsed to emphasize the different species. *Phyllium* is divided into two well-supported clades of which one has several representatives with purple coxae (clade B, Figure 2). Within clade B, two lineages are distinctly different from the previously known *P.jacobsoni* and *P.hausleithneri* and are herein described as new species: *Phylliumgardabagusi* sp. nov. and *Phylliumnisus* sp. nov. Three species exhibit purple coxae (*P.gardabagusi*, *P.hausleithneri*, and *P.nisus*) but do not form a monophyletic group. *Phylliumjacobsoni*, with white coxae coloration, is more closely related to *P.gardabagusi*, but with weak support.

### Systematics

#### Phyllium (Pulchriphyllium)

Taxon classificationAnimaliaPhasmidaPhylliidae

Griffini, 1898

94F685EC-D567-53BB-A89A-CAB8DE276ABD

##### Type species.

*Phylliumpulchrifolium* Audinet-Serville, 1838.

#### Phyllium (Pulchriphyllium) giganteum

Taxon classificationAnimaliaPhasmidaPhylliidae

Hausleithner, 1984

220F1AA5-D098-5963-8792-229641704AF6

Figure 3A–E

##### Distribution.

Malaysia: (Peninsular) Perak, Pahang, and Selangor States; (Bornean) Sarawak (Bintulu Division) and Sabah (Pensiangan) States. Indonesia: Sumatra, Bengkulu Province; (Borneo) West Kalimantan Province (Mount Bawang).

##### Range expansion.

**Figure 3.**
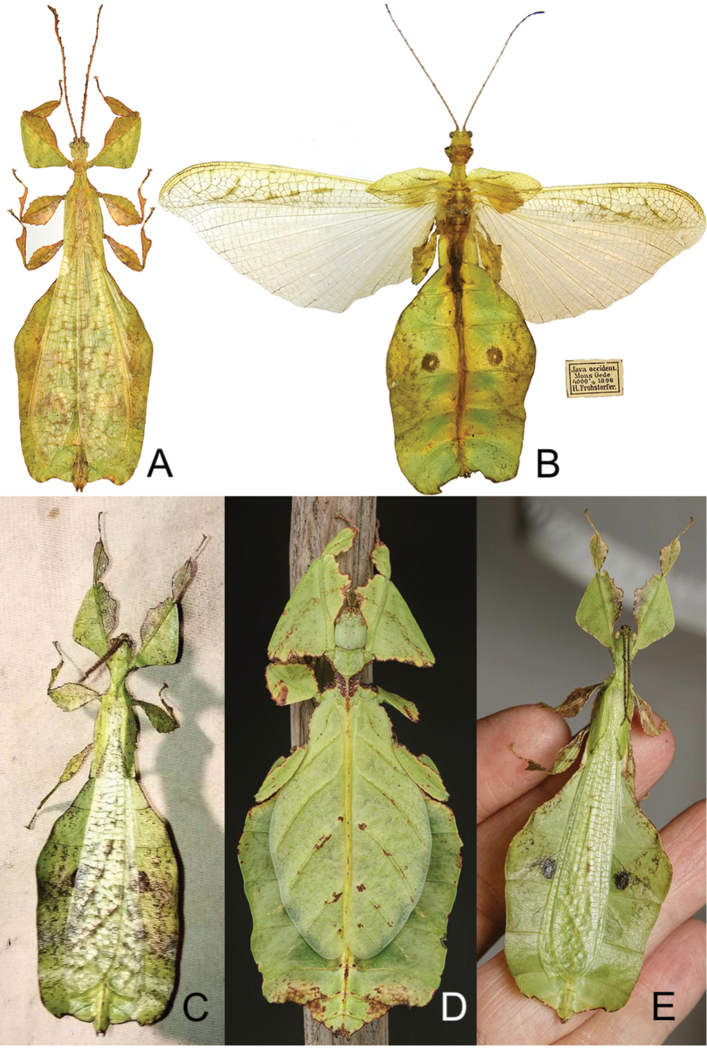
*Phylliumgiganteum*. **A, B** Dorsal view of male *Phylliumgiganteum* from Java, Indonesia **C–E** live *Phylliumgiganteum***A** male from Mount Halimun, West Java, Coll SLT**B** antique male from the MZSF collection from Mount Gede, West Java, collected by Fruhstorfer in 1898 **C** wild male photographed by Maman Cahyana (Indonesia) while out collecting at night on Mount Halimun, West Java **D** captive bred female and **E** captive bred male bred by Bruno Kneubühler, stock origin Perak, Malaysia.

Indonesia: West Java Province (Mount Halimun and Mount Gede) (Figure 3 A–C).

##### Discussion.

**Figure 4.**
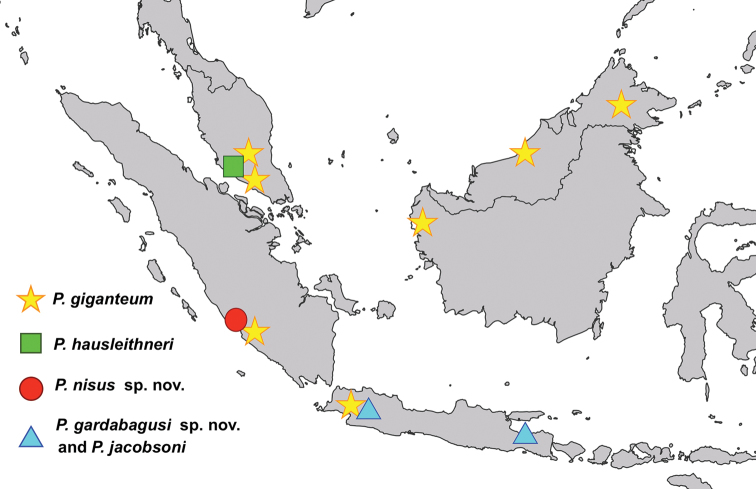
Distribution map for *Phylliumgiganteum* (Borneo, Peninsular Malaysia, Sumatra, and Java), *Phylliumhausleithneri* (Peninsular Malaysia), *Phylliumnisus* sp. nov. (Sumatra), and *Phylliumgardabagusi* sp. nov. (Java) and *Phylliumjacobsoni* (Java).

*Phylliumgiganteum* is a widely distributed species ranging from Peninsular Malaysia (the type locality), to Borneo, Sumatra, and most recently Java, Indonesia, the furthest south this species is currently known to occur (Cumming et al. 2018) (Figure 4).

As a morphologically variable species with regards to abdominal shape and coloration (Cumming et al. 2018), numerous morphological aspects have been reviewed across all known populations (including the several recently examined Javan specimens) with no consistent feature allowing morphological separation of any one population from the others. Future molecular studies based on a comprehensive taxon sampling throughout the *Phylliumgiganteum* range might provide evidence of genetic differences between populations of this widely distributed lineage. Unfortunately, given that our current review is solely based on adult morphology we have no basis to separate the widely distributed *Phylliumgiganteum* into subspecies, assuming such a distinction is warranted.

#### Phyllium (Phyllium)

Taxon classificationAnimaliaPhasmidaPhylliidae

Linnaeus, 1758

067A040B-4D79-5BEA-9493-6F5BBD9C676E

##### Type species.

Gryllis (Mantis) siccifolius Linnaeus, 1758.

#### Phyllium (Phyllium) jacobsoni

Taxon classificationAnimaliaPhasmidaPhylliidae

Rehn & Rehn, 1934

082B6639-1E46-5B76-9481-AD93FB1891A4

Figure 5A–E

##### Distribution.

Indonesia: Eastern Java (Nongkodjadjar [type locality]); Eastern Java, Mt. Argopuro; Western Java, Mt. Halimun (Figure 4).

##### Discussion.

With the description of the very morphologically similar *Phylliumgardabagusi* sp. nov., also from Java, the numerous localities noted within Hennemann et al. (2009) cannot be confirmed as *Phylliumjacobsoni*. This is because the only clear morphological feature to differentiate between these two populations is the coxae color, which can fade in specimens which were not well preserved. Therefore, we only list the localities where we have been able to confirm specimens as *Phylliumjacobsoni*. Nevertheless, with this species confirmed from both West and East Java, it is likely the species is found throughout the island.

##### Differentiation.

**Figure 5.**
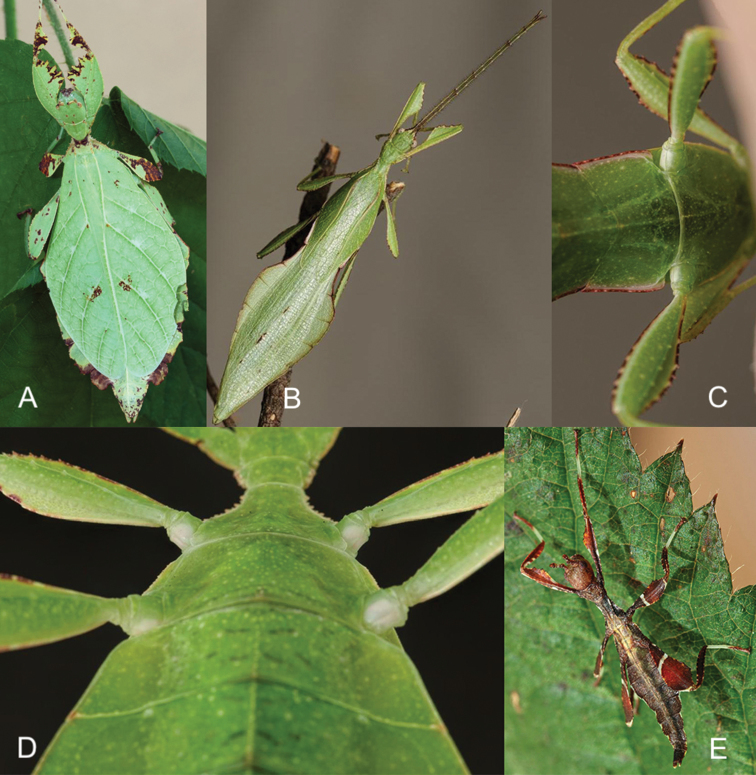
*Phylliumjacobsoni* live captive bred individuals, photos courtesy of Bruno Kneubühler. **A** Female **B** male **C** male ventral view of coxae **D** female ventral view of coxae **E** freshly hatched nymph.

This is the only species in the clade that can easily be differentiated morphologically from other clade members. The coxae color in *P.jacobsoni* is white (see Figure 5C, D) compared to a distinct and dark purple in the other three clade members (Table 1). This purple color is easier to view in females and can only be faintly seen on very well preserved or live males. In any discolored male it is impossible to differentiate the species by looking at the coxae because the white and faint purple color do not preserve well and cannot be differentiated on poor quality specimens. It is fortunate that the holotype for *Phylliumjacobsoni* is a female specimen as it can clearly be seen on the holotype female that this was not the sympatric species *Phylliumgardabagusi* sp. nov. with purple coxae and is instead a specimen with white coxae which defines *Phylliumjacobsoni*.

On average *P.jacobsoni* tend to be smaller individuals, but as with the other species in the clade there were significant outliers that made the range of sizes overlap with the other species significantly (Table 1). *Phylliumjacobsoni* newly hatched nymphs (Figure 5E) appear to be the most variable in coloration of the four clade B members. *Phylliumjacobsoni* overall coloration ranges from reddish brown to dark brown (in the other three members dark brown to black), and on *P.jacobsoni* the white transverse band on the meso- and metafemora can be a solid line (like in the other three clade B species) or can occasionally be a broken white line (but this is a less common form).

**Table 1.** Morphological features compared between the members of clade B. Key: ^a^ As noted in Hennemann et al. (2009). ^b^ 75 mm for *Phylliumjacobsoni* is noted from personal communication with Bruno Kneubühler who bred *P.jacobsoni* from the type locality of Nongkodjadjar, East Java. ^c^Größer (2008) lists *P.hausleithneri* as having 44–46 teeth, and Hennemann et al. (2009) note 44–48 teeth. ^d^ As noted in Cumming et al. (2018). ^e^ Only counted on paratype specimens Coll RC 16-203 and Coll RC 18-418.

Feature	*Phylliumjacobsoni*	*Phylliumhausleithneri*	*Phylliumnisus* sp. nov.	*Phylliumgardabagusi* sp. nov.
Distribution	Java	Peninsular Malaysia	Sumatra	Java
Female length [mm]	63.5–75.0^a/b^	74.6–82.8	70.3–79.3	68.4–77.3
Male length [mm]	42.5–56.5^a^	55.8–57.8	51.4–56.1	50.0–50.5
Number of teeth on the *pars stridens* of antennomere III in females	40^a^	44–48^c^	37–44^d^	34–39^e^
Egg color	Tan to medium brown	Dark, rich brown	Pale tan to medium brown	Pale brown to medium brown
Coxae color	White	Purple	Purple	Purple

#### Phyllium (Phyllium) hausleithneri

Taxon classificationAnimaliaPhasmidaPhylliidae

Brock, 1999

42794F81-DB83-51F4-96BA-B87AD2FE3E4C

Figure 6A–E

##### Distribution.

Malaysia: (Peninsular) Perak and Pahang states (Figure 4).

Unconfirmed distributions:

Malaysia: Selangor State, Bukit Kutu. This record was noted as *Phylliumsiccifolium* in Brock (1999) and treated as a smooth abdominal form of *P.hausleithneri* in Hennemann et al. (2009), however, without examining the specimen in question it is best to leave this as an unconfirmed record. This is because the morphologically similar *Phylliumrubrum* Cumming, Le Tirant, & Teemsma, 2018 is also found in Peninsular Malaysia and has in the past been considered a form of *P.hausleithneri*. *Phylliumrubrum* can be differentiated from *Phylliumhausleithneri* by the larger size (90.0 mm or larger in female *P.rubrum* and 74.6–82.8 mm in *P.hausleithneri*) and coxae color (red/orange in *P.rubrum* and purple in *P.hausleithneri*). Brock (1999) lists this Selangor State record as *Phylliumsiccifolium* and notes that females from Peninsular Malaysia are ranging in size from 77.0 to 92.0 mm in length which encompasses the range of sizes of both these species. Therefore, without examining the specimen from Bukit Kutu, we must treat this identification as tentative until it can be confirmed as *P.hausleithneri* or *P.rubrum*.

Singapore: Seow-Choen (2017) listed this species, but gave no actual specimen records, and his included photos were stated as coming from Tapah Hills, Perak, Malaysia, so we are unsure of the basis for the inclusion. He could have been referencing to a specimen listed in Klante (1976) from Singapore (“Bukit Timah rd.”; incorrectly identified as *Phylliumwoodi* Rehn & Rehn, 1934), which Klante noted as being 75.0 mm in length, matching the size range of *P.hausleithneri* and not *P.rubrum*. But with so many errors in identification over the years we do not include this record in our confirmed distribution for this species yet and wait until additional material is collected and confirmed from Singapore.

##### Discussion.

**Figure 6.**
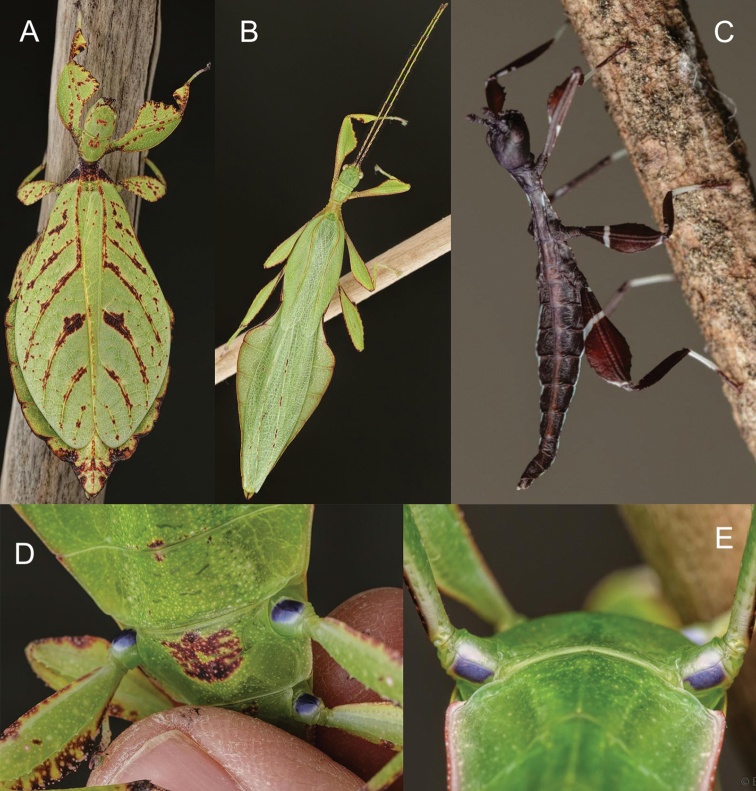
*Phylliumhausleithneri* live captive bred individuals, photos courtesy of Bruno Kneubühler. **A** Female **B** male **C** freshly hatched nymph **D** female ventral view of purple coxae **E** male ventral view of purple coxae.

This species has been in the phasmid breeding community for many years (Figure 6A–E), with most of those years being sold erroneously as *Phylliumsiccifolium* which has led to much of the confusion surrounding this species. To help clear up confusion, the morphological variations and numerous misidentifications were presented in the discussion of *Phylliumhausleithneri* in Hennemann et al. (2009). *Phylliumhausleithneri* from Peninsular Malaysia was morphologically compared extensively to *P.nisus* sp. nov. in Cumming et al. (2018) before it was realized that *P.nisus* sp. nov. was an undescribed sister species. Newly hatched nymphs of *P.hausleithneri* (Figure 6C) cannot be distinguished from the two new species from within clade B described below as their coloration falls within their observed variation.

### Description of new species

#### Phyllium (Phyllium) nisus sp. nov.

Taxon classificationAnimaliaPhasmidaPhylliidae

5A03C61F-E19C-5206-B0BB-9C68AA180740

http://zoobank.org/7FE8BB94-1E94-4337-BA9C-3DA2A1224DFF

**Figure 7.**
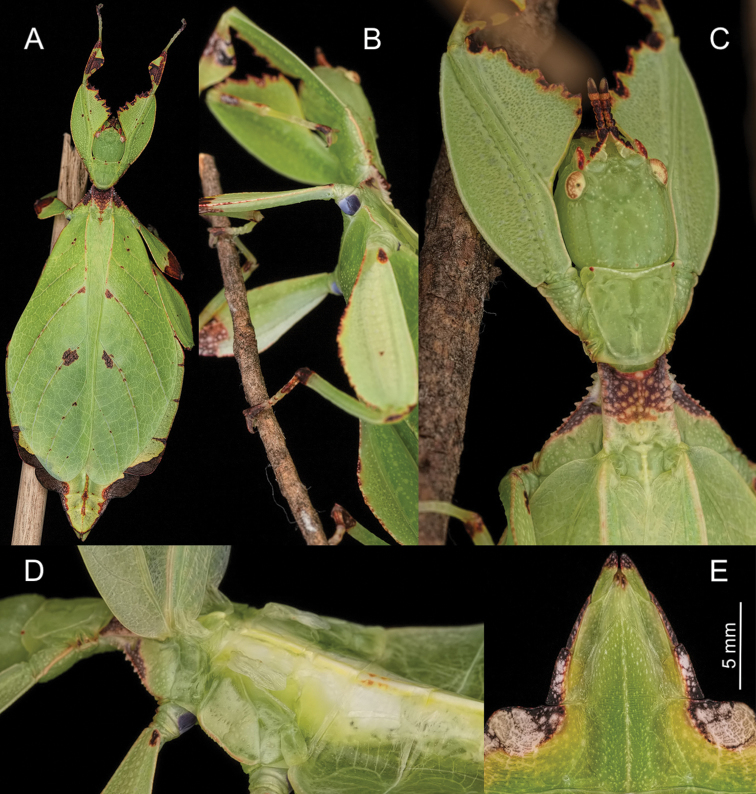
Female *Phylliumnisus* sp. nov. live captive bred individuals, photos courtesy of Bruno Kneubühler. **A** dorsal view **B** ventral view of purple coxae **C** dorsal head and thorax details **D** tegmina held open to show exposed underdeveloped alae **E** ventral genitalia details.

**Figure 8.**
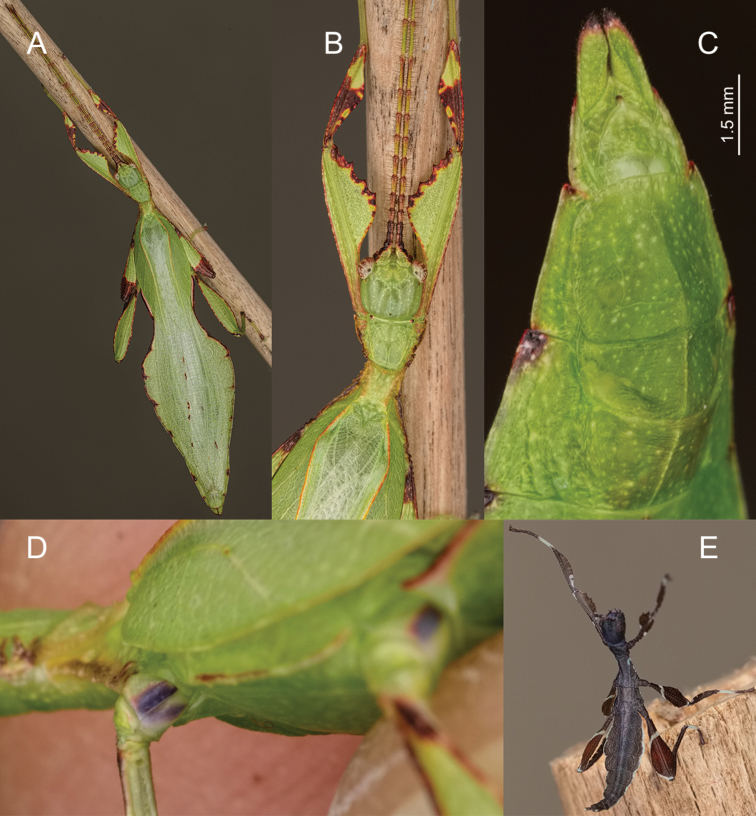
Male *Phylliumnisus* sp. nov. live captive bred individuals, photos courtesy of Bruno Kneubühler. **A** Dorsal view **B** dorsal head and thorax details **C** ventral genitalia details **D** ventral view of purple coxae **E** freshly hatched nymph.

**Figure 9.**
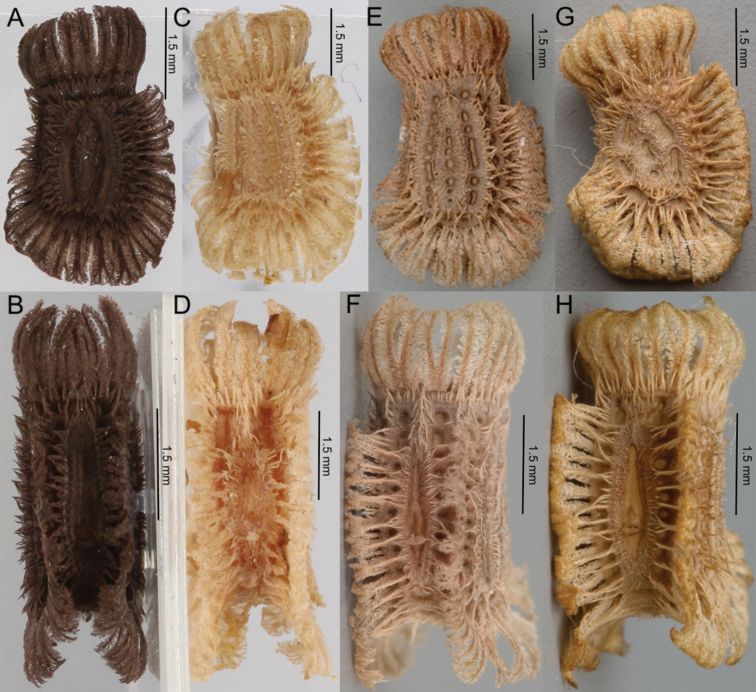
Eggs from the four species in clade B. **A***Phylliumhausleithneri*, lateral view (Coll RC 18-002) **B** micropylar plate view (Coll RC 18-003), note that this individual exhibits a slightly longer micropylar plate than average which makes it appear longer than the other clade members, this is simply the upper limit of the morphological variation **C***Phylliumnisus* sp. nov., lateral view (Coll RC 17-380) **D** micropylar plate view (Coll RC 17-377) **E***Phylliumgardabagusi* sp. nov., lateral view **F** micropylar plate view **G***Phylliumjacobsoni*, lateral view **H** micropylar plate view. Photos **E–H** courtesy of Bruno Kneubühler.

**Figure 10.**
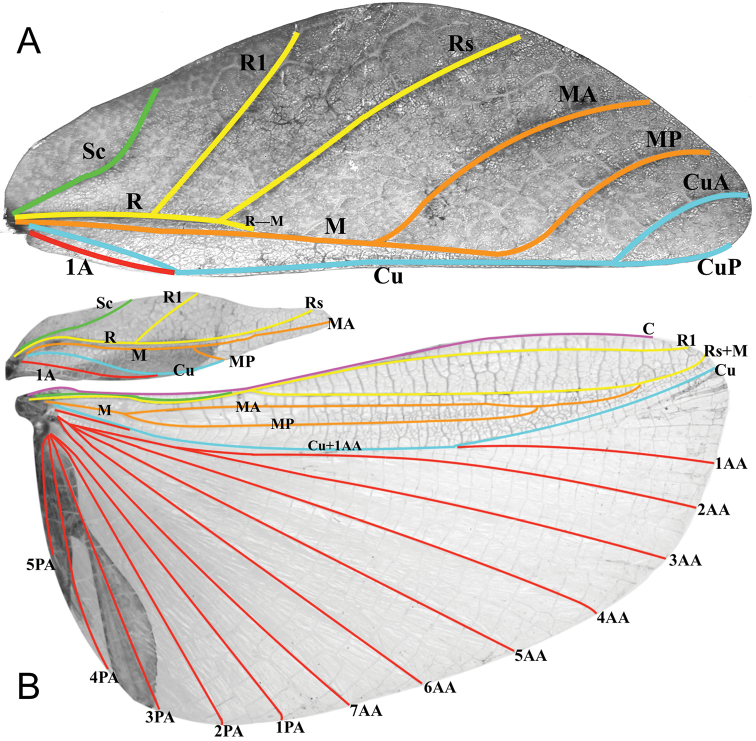
Representative female and male tegmina and alae wing venation present in our molecularly identified Clade B (see Figure 2), which is present in the new species *Phylliumgardabagusi* sp. nov. and *Phylliumnisus* sp. nov. **A** Female tegmina illustrated here is a *Phylliumnisus* sp. nov. (Coll RC 17-107) **B** male tegmina and alae illustrated here is a *Phylliumhausleithneri* (Coll RC 16-087). Abbreviations used: **C** (costa); **Sc** (subcosta); **R** (radius); **R1** (radius 1); **Rs** (radial sector); **R**–**M** (radius and medial crossvein); **M** (media); **MA** (media anterior); **MP** (media posterior); **Cu** (cubitus); **CuA** (cubitus anterior); **CuP** (cubitus posterior); **Cu+1AA** (cubitus and first anterior anal); **1A** (first anal); **1AA–7AA** (first through seventh anterior anal); **1PA–5PA** (first through fifth posterior anal).

Figures 7A–E , 8A–E , 9C–D , 10A , 13A

##### Type material.

Holotype: ♀, Indonesia: Sumatra, Bengkulu Prov., Bengkulu District, Besuki Village: IV.2017, Local Collector. Deposited in the Montreal Insectarium type collection (Coll RC 18-157) (Figure 13A). Paratypes: of 128 ♀♀, 36 ♂♂, and 39 eggs are deposited in the collections of Royce T. Cumming, Stephane Le Tirant, Oskar V. Conle, the Bogor Zoology Museum, and the Montreal Insectarium (see Suppl. material 3: Table S3 for details).

##### Discussion.

This population has been available within the phasmid breeding community for a number of years under the name *Phyllium* sp. “Bukit Daun” and has been noted as a reasonably easy species to breed in captivity (Figures 7A–E, 8A–E).

This population has already undergone extensive morphological scrutiny in Cumming et al. (2018) where no significant morphological features were identified between the Peninsular Malaysia and Sumatran populations to allow visual separation based on adults alone. The only consistent visible feature between the two populations is the color of the eggs, with *Phylliumhausleithneri* from Peninsular Malaysia having dark brown eggs (Figure 9A, B) and *Phylliumnisus* sp. nov. with pale tan eggs (Figure 9C, D). The only closely related species which can consistently be morphologically separated is *Phylliumjacobsoni* by coxae color (white in *P.jacobsoni*, Figure 5C, D, and purple in *P.nisus* sp. nov., Figures 7B, 8D). Newly hatched nymphs of *P.nisus* sp. nov. (Figure 8E) cannot be differentiated from the dark form of *P.jacobsoni* or the average *P.hausleithneri* nymphs, and their identical morphology helps to illustrate their shared common ancestry.

*Phylliumnisus* sp. nov. is possibly the species referred to in Klante (1976) from Sumatra, which he erroneously attributed to *Phylliumwoodi* Rehn & Rehn, 1934. The specimen he examined was rather large at 79.0 mm, which falls within the observed range of *Phylliumnisus* sp. nov. (see Table 1), and, based on the prominent profemoral interior lobe teeth, this specimen could be a large major form of *Phylliumnisus* sp. nov. female. However, without examining this specimen in person we are not confident in attributing this specimen to *Phylliumnisus* sp. nov. due to the cryptic nature of congenerics and therefore must leave this historic record at speculation. For full morphological measurements see Suppl. material 3: Table S3. Fortunately, a sizeable series of bred paratype specimens were examined thus allowing adequate description of the average adults and egg coloration. Coloration of the overall body was rather variable as discussed below, but the coxae and egg coloration showed little variation between individuals and we consider these colors as a reliable feature for differentiation.

##### Description.

**Female. *Coloration.*** Individuals are always a vibrant pale green with varying degrees of reddish or grayish brown coloration on specific regions of the body (Figures 1B, 7A). On the lightest colored individuals, no brown markings are present, with even the antennae a pale color similar to the shade of green on the head capsule (see the holotype female in Figure 13A for an example of a female without brown markings). In individuals which are more colored, the areas which are most frequently with these brown markings are the antennae, frontal convexity, protibiae, profemoral interior lobe, prescutum, mesopleurae (Figure 7C), small interior patches of brown on the tegmina, and the terminal abdominal segments VII–X margins (Figure 7A). On intermediate colored individuals, these features can be a third to half colored with brown, and in darker individuals these features are always at least half colored with some features almost completely colored with brown. Compound eyes are generally paler than the head capsule and usually of a yellow coloration with slight tan striping (Figure 7A). Meso- and metacoxae ventrally always with a dark blue to purple coloration which is only visible when the legs are bent forward (Figure 7B).

***Morphology.****Head.* Head capsule about as long as wide, vertex with granulation throughout the surface, some more closely spaced than others (Figure 7C). The posteromedial tubercle is broader and taller than any other nodes, on the head. Frontal convexity broad and about as long as the first antennomere, and with slight granulation on the dorsal surface and several setae present which are longer than any setae on the antennae. Compound eyes only slightly protruding from the head capsule, but are significantly large, taking up about one fourth of the length of the lateral head capsule margins (Figure 7C). Ocelli absent. Antennal fields slightly wider than and about as long as the length of the first antennomere. *Antennae.* Antennae consisting of nine segments, with the terminal segment about the same length as the preceding two segments’ lengths combined (Figure 7C). Antennomeres I–VII sparsely marked with small transparent setae, the terminal two antennomeres are covered in stout, brown setae. The *pars stridens* of antennomere III has 37–44 teeth. *Thorax.* Pronotum with gently concave anterior margin and nearly straight lateral margins, which converge to a straight posterior margin that is half the width of the anterior margin (Figure 7C). Pronotum anterior margin with small lateral defensive spray gland openings (no detectable defensive spray has been noted for these while in breeding however). The pronotum surface is marked with only minimal small granulation, with only a prominent pit in the center, and slight furrows anterior and lateral to the pit (Figure 7C). The pronotum has a prominent anterior rim and weakly formed lateral and posterior rims, all of which have only slight granulation (Figure 7C). Prosternum and the mesosternum with stout and numerous nodes, those on the anterior half of the mesosternum on the sagittal plane are slightly larger than those on the prosternum. Metasternum with short granulation throughout the entire surface. Prescutum as long as wide or occasionally slightly longer than wide, but never wider than long (Figure 7C). Lateral rims with nine to eleven lumpy node-like tubercles ranging in size from small to medium with small granulation present throughout the length interspersed with the tubercles (Figure 7C). Prescutum anterior rim prominent but not strongly protruding, with a distinct singular tubercle with the remainder of the rim relatively smooth (Figure 7C). Prescutum crest (excluding the tubercle of the anterior rim) with four to five distinct but not large nodes evenly spaced and nearly uniform in size, or with the anterior most node slightly larger than the rest. The Prescutum crest is not prominently protruding because the smooth surface of the Prescutum rises up to it, making the crest not much more than the nodes along the sagittal plane (Figure 7C). Mesopleurae beginning near the anterior margin of the Prescutum and evenly diverging; lateral margin with eight to eleven tubercles which are largest on the anterior end and steadily decreasing in size as they reach the posterior, eventually no larger than nodes (Figure 7C). Some of the largest tubercles have slightly granular surfaces or granulation at the base. Face of the mesopleurae with granulation throughout, and with two notable divots, one on the anterior third and one nearer the posterior third (Figure 7C). *Wings.* Tegmina length variable, ranging in length from halfway through abdominal segment VII to at most reaching about three quarters of the way into segment VIII. Tegmina venation is rather stable between individuals (Figure 10A). The subcosta (Sc) is the first vein in the forewing and bends inward away from the anterior margin. The radius (R) spans the central portion of the forewing with two subparallel branched veins; radius 1 (R1) terminates anterior to the widest medial expansion of the tegmina, and the radial sector (Rs) terminates posterior to the widest medial expansion, therefore the R1 and Rs occupy the majority of the center of the wing. There is a weak continuation of the radius following the prominent Rs branching which continues on as a short and thinner R–M crossvein that does not appear to solidly connect the two veins fading as it reaches the media. The media (M) is simply bifurcate with both the media anterior (MA) and media posterior (MP) terminating close to the posterior fourth of the wing. The cubitus (Cu) is also bifurcate, branching near the posterior fifth of the wing into the cubitus anterior (CuA) and cubitus posterior (CuP) which both terminate at or very near the wing posterior apex. The first anal vein (1A) is simple and fuses with the cubitus early on, only slightly past the branching distance of the R1 from R (Figure 10A). Alae rudimentary, only about 4.0 mm in length (Figure 7D). *Abdomen.* Abdominal shape quite variable, with the only consistent feature being segments II through the anterior two thirds of IV diverging, with the posterior third of segment IV the widest segment. Segments V through VIII are variable and can have perfectly straight margins (giving the abdomen a smooth spade shaped appearance, similar to the holotype female in Figure 13A) or strongly lobed margins (like in Figure 1B). Between these two extremes there are all possible forms/degrees of lobed margins with the forms continuous, not discrete. Segments IX and X are notably narrower than the previous segments and converge uniformly without lobes to the rounded apex. *Genitalia.* Subgenital plate starts at the anterior margin of segment VIII, is broad, and extends halfway to three quarters into segment X, ending in a fine point (Figure 7E). Gonapophyses are long and slender, reaching or very slightly exceeding the apex of abdominal segment X (Figure 7E). Cerci flat, not strongly cupped, with a granular surface and few detectable setae (Figure 7E). *Legs.* Profemoral exterior lobes narrow and smoothly arcing from end to end without a strongly notable angle, narrower than the width of the interior lobe (Figure 7C). Edge of the profemoral exterior lobe smooth without notable granulation or teeth (Figure 7C). Profemoral interior lobe wider than the exterior and with a right angle or slightly obtuse angle and marked with four to five teeth (Figure 7C). These teeth have a slightly wider gap in the center, and are variable in size ranging from small serrate teeth to larger angular teeth (Figure 7C). Generally, the size of the teeth is paralleled by the size of the abdominal lobes but not always. Mesofemoral exterior lobe arcs from end to end but is slightly weighted towards the distal half and marked with one to three small serrate teeth distributed on the distal half only. Interior and exterior lobes can be of similar width, or interior lobe can be slightly thinner. Mesofemoral interior lobe arcs end to end with five to six small serrate teeth only on the distal half of the arc which is slightly wider than the proximal half of the arc. Metafemoral interior lobe arcs end to end and has five to six serrate teeth on the distal half of the lobe. Metafemoral exterior lobe is thin and smooth, hugging the metafemoral shaft and generally with no teeth but occasionally with one to two at the distal most edge. Protibiae lacking an exterior lobe. Protibiae interior lobe spans the entire length of the protibiae and can be one and a half to two times the width of the protibiae shaft itself. The lobe is distinctly triangular and can be nearly evenly distributed or slightly wider on the distal half. Mesotibiae and metatibiae lacking exterior and interior lobes.

**Male. *Coloration.*** Overall coloration pale green throughout with variable patches of brown to reddish coloration (Figure 8A). Compound eyes are generally paler than the head capsule and of a yellow coloration with slight tan striping (Figure 8B). The antennae are darker in color, with each segment exhibiting a slight green in addition to brown towards the apex of each segment, which gives the antennae an overall striped appearance of green and brown (Figure 8B). Males can be completely green lacking any brown coloration except slight brown markings on the protibial interior lobe, or they can range to the other extreme with brown markings on protibial, profemoral, and mesofemoral lobes along with the margins of the metafemoral lobes and the margin of the abdomen (Figure 8A). Meso- and metacoxae ventrally with a pale to dark purple and white coloration (Figure 8D). The coloration on the mesocoxae is generally darker and easier to distinguish than the purple on the metacoxae, which is only a faint purple hue on a mostly white surface.

***Morphology.****Head.* Head capsule longer than wide, with a vertex that is nearly completely smooth or in some individuals there can be two or three small nodes near the posteromedial tubercle (Figure 8B). Frontal convexity stout with sparse thin setae. The posteromedial tubercle is not broad but is distinctly raised from the head capsule. Compound eyes large and bulbous, taking up over one third of the head capsule lateral margins (Figure 8B). Three ocelli moderately developed located between and slightly posterior to the compound eyes. Antennal fields as wide and as long as the scapus. *Antennae.* Antennae (including the scapus and pedicellus) consists of 23 segments, all segments except the scapus and pedicellus and terminal four segments are covered in dense setae that are as long as or longer than the antennae segment is wide. The terminal four segments are covered in dense short setae and the scapus and pedicellus are nearly completely bare. *Thorax.* Pronotum with anterior margin distinctly concave and lateral margins that are slightly convex and converging to a straight posterior margin that is slightly more than half the width of the anterior rim (Figure 8B). Anterior margin of the pronotum has a distinct rim, lateral margins have moderate rims, and the posterior margin lacks a rim (Figure 8B). Face of the pronotum is marked by a distinct furrow and pit in the center, a smooth surface, and three distinct pits along the anterior margin (Figure 8B). Prosternum is granulose throughout with small nodes of even size and spacing. Mesosternum surface marked with more prominent nodes, with the largest along the sagittal plane and more strongly on the anterior margin, posterior margin with less prominent and small nodes. Prescutum longer than wide, with lateral margins slightly converging to the posterior (Figure 8B). Lateral rims with eight to nine tubercles of varying size, some prominent but others rather small and not much more prominent than nodes (Figure 8B). Prescutum crest along the sagittal plane with four to five small nodes of uniform size and spaced throughout the length. The surface of the prescutum rises up to meet the crest with a face that is smooth (Figure 8B). Prescutum anterior margin marked with a tubercle, which is only about two times larger than any of the other nodes along the crest. Mesopleurae not notably wide and diverge almost uniformly along the entire length, diverging slightly more prominently at the posterior margin (Figure 8B). Lateral margin with four to five major tubercles throughout the length, and between six and nine smaller minor tubercles interspersed throughout. Each tubercle is marked by either a single stiff, short setae or with as many as three setae. Face of the mesopleurae slightly wrinkled and with two faint divots, one on the anterior third and one on the posterior third. Tegmina moderate length, extending halfway to three quarters the way through abdominal segment III. *Wings.* Tegmina wing venation (see Figure 10B for general venation found in the species of this clade): the subcosta (Sc) is the first vein and terminates the earliest, about one third of the way through the overall tegmina length. The radius (R) spans the entire length of the tegmina with the radius 1 (R1) branching just anterior to the middle and terminating just posterior to the middle of the wing with the radial sector (Rs) terminating nearly at the wing apex. The media (M) also spans the entire length of the tegmina with the media posterior (MP) branching off slightly posterior to the middle and terminating promptly. The cubitus (Cu) terminates past the midline upon meeting the media posterior. The first anal (1A) vein terminates upon reaching the cubitus near the midline. Alae well developed in an oval fan configuration, long, reaching to the anterior margin of abdominal segments IX or X. Alae wing venation (see Figure 10B for general venation found in the species of this clade): the costa (C) is present along the entire foremargin giving stability to the wing. The subcosta (Sc) is short, spanning less than a third of the wing length and is mostly fused with the radius in the beginning but terminates when it meets the costa. The radius (R) spans the entire wing and branches approximately a third of the way through into the radius 1 (R1) and radial sector (Rs) which run nearly parallel through most of their length until they terminate at the wing apex near each other but not touching. The media (M) branches early (only about a sixth of the way through the wing into the media anterior (MA) and the media posterior (MP) which run parallel with each other throughout the central two thirds of the wing until the media posterior fuses with the media anterior which eventually fuses with the above radial sector about one sixth of the way from the wing apex. The cubitus (Cu) runs unbranched and terminates at the wing apex. Of the anterior anal veins, the first anterior anal (1AA) fuses with the cubitus near the point where the media branches into the media anterior and media posterior and then the first anterior anal branches from the cubitus three fifths of the way through the wing length where it uniformly diverges from the cubitus until it terminates at the wing margin. The anterior anal veins two through seven (2AA–7AA) have a common origin and run unbranched in a folding fan pattern of relatively uniform spacing to the wing margin. The posterior anal veins (1PA–5PA) share a common origin separate from the anterior anal veins and run unbranched to the wing margin with slightly thinner spacing than the anterior anal veins. *Abdomen.* Abdominal segments II slightly converging, III through the anterior two thirds of segment IV diverging to the widest portion. The posterior of IV through V either almost parallel sided or converging, and segment VI through X uniformly converging (Figure 8A). *Genitalia.* Poculum broad, and ends in a straight margined apex that slightly passes the anterior margin of segment X (Figure 8C). Cerci long and slender, extending from under the anal abdominal segment, slightly cupped, covered in a granulose surface and numerous short setae (Figure 8C). Vomer broad and stout with straight sides evenly converging, and a thick single apical hook which hooks upwards into the paraproct (Figure 8C). *Legs.* Profemoral exterior lobe one third to two thirds the width of the interior lobe, hugging the curve of the profemoral shaft and marked with a granular margin and fine small setae but no notable teeth (Figure 8B). Profemoral interior lobe roundly triangular and marked with five teeth which can be small and serrate or larger and triangular in more prominent individuals (Figure 8B). Mesofemoral exterior lobe arcs end to end, but is significantly weighted on the distal half which is marked with one to two serrate teeth, and the proximal half that is rather thin. Mesofemoral interior lobe is slightly thinner than the exterior lobe, is broader on the distal end and is marked with five to six small serrate teeth. Metafemoral exterior lobe lacks dentition, and has a straight margin along the metafemoral shaft. Metafemoral interior lobe smoothly arcs end to end with seven to eight small serrate teeth on the distal half. Protibiae lacking exterior lobe, interior lobe reaching end to end in a smooth evenly weighted triangle only one to one and a half times as wide as the protibial shaft (Figure 8B). Meso- and metatibiae simple, lacking lobes completely.

##### Eggs.

The lateral surfaces are flattened and the dorsal surface is slightly convex, which gives the egg a slight bend (Figure 9C, D). When viewed from the lateral aspect, the anterior width of the capsule is the narrowest, with the width slightly increasing steadily to the posterior, but only slightly so (Figure 9C). When viewed from the lateral aspect, the dorsal margin has long feather-like pinnae with single or double branching tips along almost the entire length with occasionally some individuals with the anterior most area lacking these long pinnae, the posterior margin also has these long pinnae (Figure 9C). The ventral margin lacks these long feather-like pinnae on the edges, but instead has a row of slightly shorter pinnae along the posterior half of the ventral surface sagittal plane with those at the posterior the largest followed by pinnae steadily decreasing in length as they reach the anterior which lacks these sagittal pinnae (Figure 9C). Lateral surfaces with three rows of bald impressions, with the space between densely covered with short carpet-like pinnae (Figure 9C). These three rows of impressions are variable between individuals, but the most common is that the three bald rows are continuous, not broken into smaller bald impressions, however, some individuals have been observed as having the bald impressions slightly broken up into smaller portions. Micropylar plate ranging from two thirds to four fifths of the overall dorsal surface length, with the thickest portion the center or slightly off center towards the micropylar cup (Figure 9D). Micropylar plate teardrop shaped, with most of the width as wide as the micropylar cup (Figure 9D). Micropylar cup of moderate size and placed on the posterior quarter of the capsule (Figure 9D). Operculum slightly ovular, with the outer margin with a row of pinnae similar to those along the posterior edge of the capsule, rarely forked, almost always with a single prominent end. Overall color light tan (Figure 9C, D).

Measurements including the extended pinnae [mm]. Length (including operculum) 6.2–6.5 mm, maximum width of capsule when viewed from lateral aspect 3.6–3.8 mm, length of micropylar plate 2.5–2.6 mm.

##### Newly hatched nymphs.

General color throughout the body (including head and antennae) is dark brown to black (Figure 8E). Margins of the abdomen are white. Meso- and metafemora with a continuous transverse white band on the proximal third; a small white spot on the interior lobe proximal margin; and a thin white crescent on the distal exterior lobe margin. Profemora dark brown to black, except for near the proximal third where there can be small white spots on each side of the profemoral shaft, but not a solid transverse white band. Tibiae on all legs the same dark brown to black as the rest of the body but with faint two to three tan spots on all of the interior margins on the proximal third, or more clearly white than tan on the protibial interior lobe. Basitarsi are white and remaining tarsal segments are tan to dark brown.

##### Etymology.

Noun, Greek in origin, Νῖσος. Named after Nisus, king of Megara, who had a single purple lock of hair that, for as long as it was not cut, guaranteed him life and possession of his kingdom. We felt that this homage was fitting to the purple-haired king as this species has the singular purple feature (coxae) which is unique among the Phylliidae with only the species in this clade known to have purple coxae.

#### Phyllium (Phyllium) gardabagusi sp. nov.

Taxon classificationAnimaliaPhasmidaPhylliidae

3019A0D4-483E-54E1-B106-370F8CC86607

http://zoobank.org/81385867-BCE0-4D79-8372-3CF5A9E5C32C

**Figure 11.**
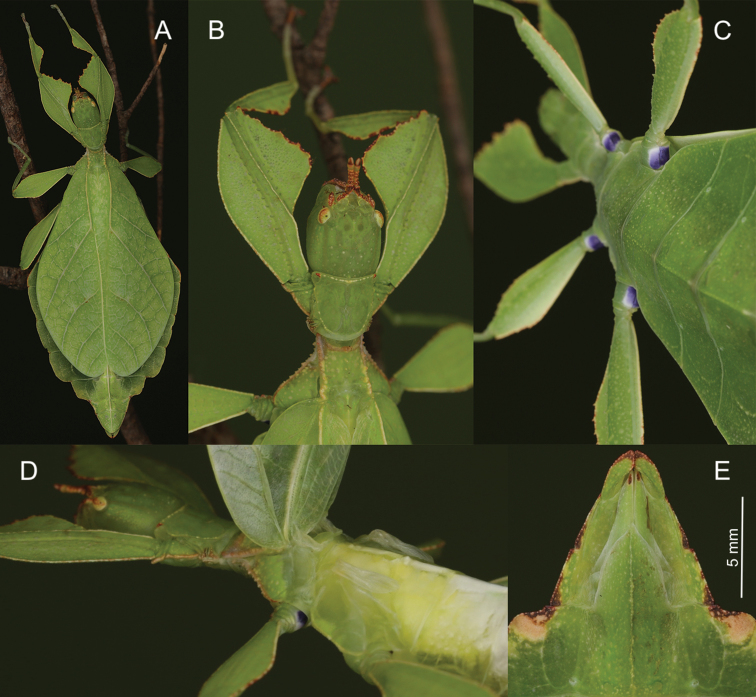
Female *Phylliumgardabagusi* sp. nov. live captive bred individuals, photos courtesy of Bruno Kneubühler. **A** Dorsal view **B** dorsal head and thorax details **C** ventral view of purple coxae **D** tegmina held open to show exposed underdeveloped alae **E** ventral genitalia details.

**Figure 12.**
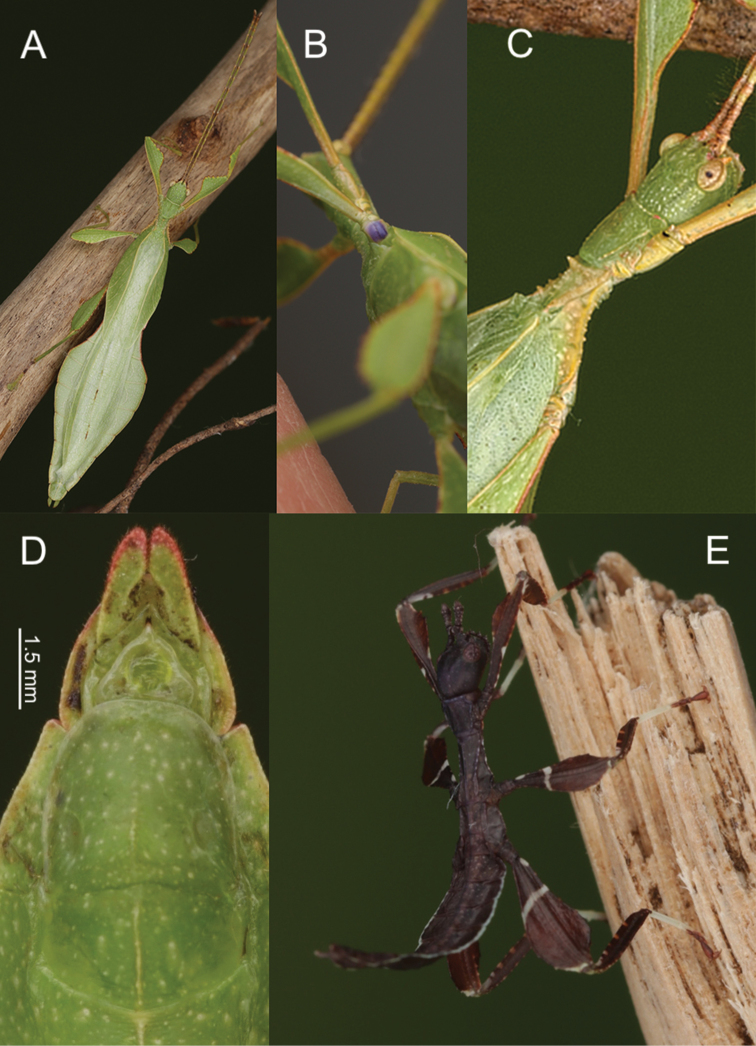
Male *Phylliumgardabagusi* sp. nov. live captive bred individuals, photos courtesy of Bruno Kneubühler. **A** Dorsal view **B** ventral view of purple coxae **C** dorsolateral, head and thorax details **D** ventral genitalia details **E** freshly hatched nymph.

**Figure 13.**
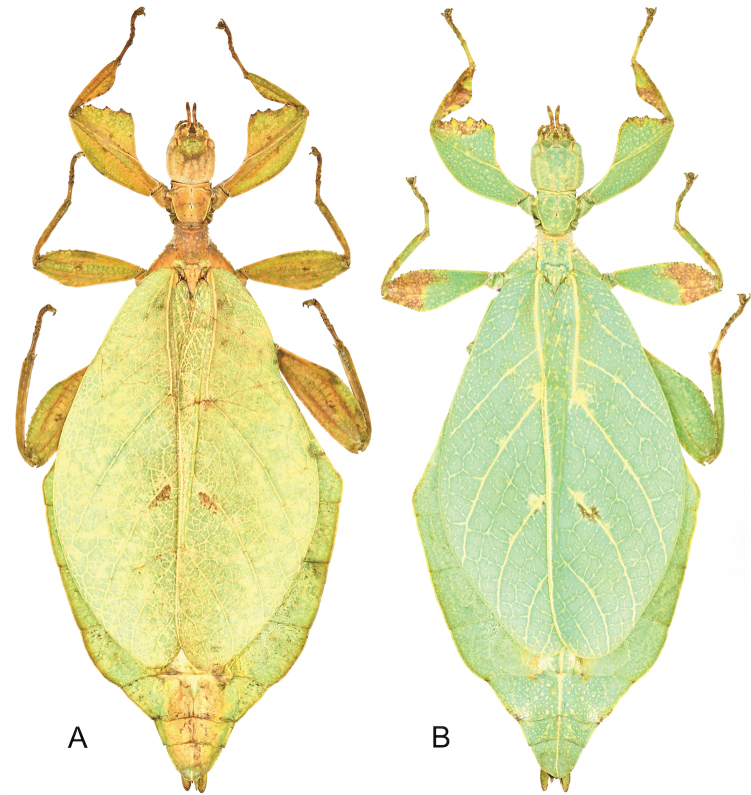
Holotypes for the two new *Phyllium* species described herein. **A**Phyllium (Phyllium) nisus sp. nov. from Sumatra, Indonesia **B**Phyllium (Phyllium) gardabagusi sp. nov. from Java, Indonesia.

Figures 9 E, F , 11 A–E , 12 A–E , 13B

##### Type material.

Holotype: ♀: Indonesia: West-Java, Mt. Halimun: August 2014. Deposited in the Montreal Insectarium type collection (Coll RC 16-202) (Figure 13B). Paratypes of 23 ♀♀, 18 ♂♂, and 50 eggs are deposited in the collections of Royce T. Cumming, Stephane Le Tirant, Oskar V. Conle, and Maxime Oritz (see Suppl. material 3: Table S3 for details).

##### Discussion and differentiation.

This population has also recently entered the phasmid breeding community under the culture name of *Phyllium* sp. “Argopuro, Blue-coxae” (Figures 11 A–E, 12 A–E). This was another population which the authors examined extensively looking for consistent morphological differences but due to the significant intraspecies variation within all members of this clade, no useful feature could be found to separate *P.gardabagusi* sp. nov. from either *P.hausleithneri* or *P.nisus* sp. nov. The only closely related species which can consistently be morphologically separated is *Phylliumjacobsoni* because of the coxae color (white in *P.jacobsoni* Figure 5C, D and purple in *P.gardabagusi* sp. nov. Figures 11C, 12B). For full morphological measurements see Suppl. material 4: Table S4. Fortunately, a sizeable series of bred paratype specimens were examined thus allowing adequate description of the average adults and egg coloration. Coloration of the overall body was rather variable as discussed below, but the coxae and egg coloration showed little variation between individuals and we feel these colors are a reliable feature for differentiation. Newly hatched nymphs of *P.gardabagusi* sp. nov. (Figure 12E) cannot be differentiated from dark form *P.jacobsoni* or normal *P.hausleithneri* nymphs, and their identical morphology helps to illustrate their shared common ancestry.

##### Description.

**Female. *Coloration.*** Specimens are always a vibrant pale green with varying degrees of reddish or grayish brown coloration more common on specific regions of the body. On the lightest colored individuals, no brown markings are present, with even the antennae a pale color similar to the shade of green seen on the head capsule (Figure 11A). Some lightly colored individuals have brown antennae and slight brown margins on the profemoral and protibial interior lobes and the thorax, with little to no brown coloration on the rest of the individual (Figure 1C). In individuals which are more colored, protibial interior lobes, profemoral interior lobes, mesofemoral lobes, and metatibial lobes can be a third to half colored with brown (Figure 13B). No individuals with strong brown coloration have been seen so far, but with such variable individuals it would not be surprising if this species also had individuals with darker brown coloration. Compound eyes are generally paler than the head capsule and usually of a yellow coloration with slight tan striping (Figure 11B). Meso- and metacoxae ventrally always with a royal purple coloration with a white margin which can only be viewed when the legs are bent forward (Figure 11C).

***Morphology.****Head.* Head capsule about as long as wide, vertex with granulation throughout the surface, some more closely spaced than others (Figure 11B). The posteromedial tubercle is broader and taller than any other nodes, on the head. Frontal convexity broad and about as long as the first antennomere, and with slight granulation on the dorsal surface and several setae present which are longer than any setae on the antennae. Compound eyes only slightly protruding from the head capsule, but are significantly large, taking up about one fourth of the length of the lateral head capsule margins (Figure 11B). Ocelli absent. Antennal fields slightly wider than and about as long as the length of the first antennomere. *Antennae.* Antennae consisting of nine segments, with the terminal segment about the same length as the preceding segment or slightly longer (Figure 11B).​ Antennomeres I–VII sparsely marked with small transparent setae, the terminal two antennomeres are covered in stout, brown setae.​ The *pars stridens* of antennomere III on examined paratypes have 34–39 teeth. *Thorax.* Pronotum with gently concave anterior margin and nearly straight lateral margins, which converge to a straight posterior margin that is half the width of the anterior margin (Figure 11B). Pronotum anterior margin with small lateral defensive spray gland openings (no detectable defensive spray has been noted for these while in breeding) (Figure 11B). The pronotum surface is marked with only minimal small granulation, with only a prominent pit in the center, and slight furrows anterior and lateral to the pit (Figure 11B). The pronotum has a prominent anterior rim and weakly formed lateral and posterior rims, all of which have only slight granulation (Figure 11B). Prosternum and the mesosternum with stout and numerous nodes, those on the anterior half of the mesosternum on the sagittal plane are slightly larger than those on the prosternum. Metasternum with short granulation throughout the entire surface. Prescutum as long as wide or occasionally slightly longer than wide, but never wider than long (Figure 11B). Lateral rims with eight to eleven lumpy node-like tubercles ranging in size from small to medium with small granulation present throughout the length interspersed with the tubercles (Figure 11B). Prescutum anterior rim prominent but not strongly protruding, with a distinct singular tubercle with the remainder of the rim relatively smooth or occasionally with slight granulation (Figure 11B). Prescutum crest (excluding the tubercle of the anterior rim) with four to five distinct but not large nodes evenly spaced and nearly uniform in size or with the anterior most node slightly larger than the rest (Figure 11B). Prescutum crest is not prominently protruding because the smooth surface of the prescutum rises up to it, making the crest not much more than the nodes along the sagittal plane (Figure 11B). Mesopleurae beginning near the anterior margin of the prescutum and evenly diverging; lateral margin with eight to eleven tubercles which are largest on the anterior end and steadily decreasing in size as they reach the posterior, eventually no larger than nodes (Figure 11B). Some of the largest tubercles have slightly granular surfaces or granulation at the base (Figure 11B). Face of the mesopleurae with granulation throughout, and with two notable divots, one on the anterior third and one nearer the posterior third. *Wings.* Tegmina length variable, ranging from halfway through abdominal segment VII to at most reaching about one third of the way into segment VIII. Tegmina venation is rather stable between individuals (see Figure 10A for an example of the venation found in this species). The subcosta (Sc) is the first vein in the forewing and bends inward away from the anterior margin. The radius (R) spans the central portion of the forewing with two subparallel branched veins; radius 1 (R1) terminates anterior to the widest medial expansion of the tegmina, and the radial sector (Rs) terminates posterior to the widest medial expansion, therefore the R1 and Rs occupy the majority of the center of the wing. There is a weak continuation of the radius following the prominent Rs branching which continues on as a short and thinner R–M crossvein that does not appear to solidly connect the two veins fading as it reaches the media. The media (M) is simply bifurcate with both the media anterior (MA) and media posterior (MP) terminating close to the posterior fourth of the wing. The cubitus (Cu) is also bifurcate, branching near the posterior fifth of the wing into the cubitus anterior (CuA) and cubitus posterior (CuP) which both terminate at or very near the wing posterior apex. The first anal vein (1A) is simple and fuses with the cubitus early on, only slightly past the branching distance of the R1 from R. Alae rudimentary, only about 4.0 mm in length (Figure 11D). *Abdomen.* Abdominal shape quite variable, with the only consistent feature being segments II through the anterior two thirds of IV diverging, with the posterior third of segment IV the widest segment. Segments V through VIII are variable and can have perfectly straight margins (giving the abdomen a smooth spade shaped appearance, Figure 13B) or lobed margins (Figure 11A). Between these two extremes there are all possible forms/degrees of lobed margins with the forms continuous, not discrete. Segments IX and X are notably narrower than the previous segments and converge uniformly without lobes to the rounded apex. *Genitalia.* Subgenital plate starts at the anterior margin of segment VIII, is broad, and extends halfway to three quarters into segment X, ending in a fine point (Figure 11E). Gonapophyses are long and slender, reaching or very slightly exceeding the apex of abdominal segment X (Figure 11E). Cerci flat, not strongly cupped, with a granular surface and few detectable setae (Figure 11E). *Legs.* Profemoral exterior lobes narrow and smoothly arcing from end to end without a strongly notable angle, narrower than the width of the interior lobe (Figure 11B). Edge of the profemoral exterior lobe smooth without notable granulation or teeth (Figure 11B). Profemoral interior lobe wider than the exterior and with a right angle or slightly obtuse angle and marked with four to five teeth (Figure 11B). These teeth have a slightly wider gap in the center, and are variable in size ranging from small serrate teeth to larger angular teeth (Figure 11B). Generally, the size of the teeth is paralleled by the size of the abdominal lobes but not always. Mesofemoral exterior lobes arc from end to end but are slightly weighted towards the distal half and marked with one to three small serrate teeth distributed on the distal half only. Interior and exterior lobes can be of a similar width, or interior lobe can be slightly thinner. Mesofemoral interior lobe arcs end to end with five to six small serrate teeth only on the distal half of the arc which is slightly wider than the proximal half of the arc. Metafemoral interior lobe arcs end to end and has five to six serrate teeth on the distal half of the lobe. Metafemoral exterior lobe is thin and smooth, hugging the metafemoral shaft and generally with no teeth but occasionally with one to two at the distal most edge. Protibiae lacking an exterior lobe. Protibial interior lobe spans the entire length of the protibiae and can be one and a half to two times the width of the protibiae shaft itself. The lobe is distinctly triangular, and the lobe can be evenly distributed on the proximal and distal halves or the lobe can be slightly wider on the distal half. Mesotibiae and metatibiae lacking exterior and interior lobes.

**Male. *Coloration.*** Overall coloration pale green throughout with some veins and nodes a lighter yellow color (Figure 12A). Compound eyes are generally paler than the head capsule and usually of a yellow coloration with slight tan striping (Figure 12C). The antennae are darker in color, with each segment exhibiting a slight green in addition to brown towards the apex of each segment, which gives the antennae an overall striped appearance of green and brown. Nearly all observed males were completely green lacking any brown coloration except occasionally slight brown markings on the protibial interior lobe. The only consistent brown feature was the margin of abdominal segments II–IV, and the margins of the mesofemoral lobes which have a tan to brown color. Meso- and metacoxae ventrally with a pale purple and white coloration (Figure 12B). The coloration on the mesocoxae is generally darker and easier to distinguish than the purple on the metacoxae, which is only a faint purple hue on a mostly white surface.

***Morphology.****Head.* Head capsule longer than wide, with a vertex that can be nearly completely smooth with two or three small nodes near the posteromedial tubercle or with slight granulation throughout the surface (Figure 12C). Frontal convexity with thin pale setae. The posteromedial tubercle is not broad but is distinctly raised from the head capsule. Compound eyes large and bulbous, taking up over one third of the head capsule lateral margins (Figure 12C). Three ocelli moderately developed located between and slightly posterior to the compound eyes. Antennal fields as wide and as long as the scapus. *Antennae.* Antennae (including the scapus and pedicellus) consists of 23 segments, all segments except the scapus and pedicellus and terminal four segments are covered in dense setae that are as long as or longer than the antennae segment is wide. The terminal four segments are covered in dense short setae and the scapus and pedicellus are nearly completely bare. *Thorax.* Pronotum with anterior margin distinctly concave and lateral margins that are slightly convex and converging to a straight posterior margin that is slightly more than half the width of the anterior rim (Figure 12C). Anterior margin of the pronotum has a distinct rim, lateral margins have moderate rims, and the posterior margin lacks a rim (Figure 12C). Face of the pronotum is marked by a distinct furrow and pit in the center, a smooth surface, and three distinct pits along the anterior margin (Figure 12C). Prosternum is granulose throughout with small nodes of even size and spacing. Mesosternum surface marked with more prominent nodes, with the largest along the sagittal plane and more strongly on the anterior margin, posterior margin with less prominent and small nodes. Prescutum longer than wide, with lateral margins slightly converging to the posterior (Figure 12C). Lateral rims with eight to nine tubercles of varying size, some prominent but other rather small and not much more prominent than nodes (Figure 12C). Prescutum crest along the sagittal plane with three to four small nodes of uniform size and spaced throughout the length (Figure 12C). The surface of the prescutum rises up to meet the crest with a face that is smooth. Prescutum anterior margin marked with a tubercle, which is only about two times larger than any of the other nodes along the crest (Figure 12C). Mesopleurae not notably wide and diverge almost uniformly along the entire length, diverging slightly more prominently at the posterior margin (Figure 12C). Lateral margin with five to six major tubercles throughout the length, and between five and seven smaller minor tubercles interspersed throughout (Figure 12C). Each tubercle is marked by either a single stiff, short setae or with as many as three setae. Face of the mesopleurae slightly wrinkled and with two faint divots, one on the anterior third and one on the posterior third (Figure 12C). *Wings.* Tegmina moderate length, extending three quarters the way through abdominal segment III. Tegmina wing venation (see Figure 10B for general venation found in the species of this clade): the subcosta (Sc) is the first vein and terminates the earliest, about one third of the way through the overall tegmina length. The radius (R) spans the entire length of the tegmina with the radius 1 (R1) branching just anterior to the middle and terminating just posterior to the middle of the wing with the radial sector (Rs) terminating nearly at the wing apex. The media (M) also spans the entire length of the tegmina with the media posterior (MP) branching off slightly posterior to the middle and terminating promptly. The cubitus (Cu) terminates past the midline upon meeting the media posterior. The first anal (1A) vein terminates upon reaching the cubitus near the midline. Alae well developed in an oval fan configuration, long, reaching to the anterior margin of abdominal segment X. Alae wing venation (see Figure 10B for general venation found in the species of this clade): the costa (C) is present along the entire foremargin giving stability to the wing. The subcosta (Sc) is short, spanning less than a third of the wing length and is mostly fused with the radius in the beginning but terminates when it meets the costa. The radius (R) spans the entire wing and branches approximately a third of the way through into the radius 1 (R1) and radial sector (Rs) which run nearly parallel through most of their length until they terminate at the wing apex near each other but not touching. The media (M) branches early (only about a sixth of the way through the wing into the media anterior (MA) and the media posterior (MP) which run parallel with each other throughout the central two thirds of the wing until the media posterior fuses with the media anterior which eventually fuses with the above radial sector about one sixth of the way from the wing apex. The cubitus (Cu) runs unbranched and terminates at the wing apex. Of the anterior anal veins, the first anterior anal (1AA) fuses with the cubitus in line with the branching of the media into the media anterior and media posterior and then the first anterior anal branches from the cubitus three fifths of the way through the wing length where it uniformly diverges from the cubitus until it terminates at the wing margin. The anterior anal veins two through seven (2AA–7AA) begin from a common origin and run unbranched in a folding fan pattern of relatively uniform spacing to the wing margin. The posterior anal veins (1PA–5PA) share a common origin separate from the anterior anal veins and run unbranched to the wing margin with slightly thinner spacing than the anterior anal veins. *Abdomen.* Abdominal segments II slightly converging, III through the anterior two thirds of segment IV diverging to the widest portion (Figure 12A). The posterior of IV through V either almost parallel sided or converging, and segment VI through X uniformly converging (Figure 12A). *Genitalia.* Poculum broad and ends in a straight margined apex that slightly passes the anterior margin of segment X (Figure 12D). Cerci long and slender, extending from under the anal abdominal segment, slightly cupped, covered in a granulose surface, and numerous short setae (Figure 12D). Vomer broad and stout with straight sides evenly converging, and a thick single apical hook which hooks upwards into the paraproct (Figure 12D). *Legs.* Profemoral exterior lobe one third the width of the interior lobe, hugging the curve of the profemoral shaft and marked with a granular margin and fine small setae but no notable teeth. Profemoral interior lobe roundly triangular and marked with five serrate teeth with a larger gap between the middle two. Mesofemoral exterior lobe arcs end to end but is significantly weighted on the distal half which is marked with one to two serrate teeth, and the proximal half that is rather thin. Mesofemoral interior lobe is slightly thinner than the exterior lobe, is broader on the distal end and is marked with five to six small serrate teeth. Metafemoral exterior lobe lacks dentition, and with a straight margin along the metafemoral shaft. Metafemoral interior lobe smoothly arcs end to end with eight to nine small serrate teeth on the distal half. Protibiae lacking exterior lobe, interior lobe reaching end to end in a smooth evenly weighted triangle only one to one and a half times as wide as the protibial shaft. Meso- and metatibiae simple, lacking lobes completely.

##### Eggs.

The lateral surfaces are flattened and the dorsal surface is slightly convex, which gives the egg a slight bend (Figure 9E). When viewed from the lateral aspect, the anterior width of the capsule is the narrowest, with the width slightly increasing steadily to the posterior, but only slightly so (Figure 9E). When viewed from the lateral aspect; the dorsal margin has long feather-like pinnae with single or double branching tips along three quarters to almost the entire length of the margin, with the anterior quarter occasionally lacking these long pinnae, the posterior margin also has these long pinnae (Figure 9E). The ventral margin lacks these long feather-like pinnae on the edges, but instead has a row of slightly shorter pinnae along the ventral surface sagittal plane with those at the posterior the largest followed by pinnae steadily decreasing in length as they reach the anterior which culminates in a narrow area at the anterior without pinnae near the operculum (Figure 9E). Lateral surfaces with three rows of bald impressions, with the space between densely covered with short carpet-like pinnae (Figure 9E). These three rows of impressions are variable between individuals, ranging from broken into numerous small patches, or with a majority of each impression continuous throughout its length (Figure 9E). Micropylar plate ranging from two thirds to four fifths of the overall dorsal surface length, with the thicker end situated towards the posterior half (Figure 9F). Micropylar plate in a slight teardrop shape, with most of the width as wide as the micropylar cup (Figure 9F). Micropylar cup of moderate size and placed on the posterior quarter of the capsule (Figure 9F). Operculum slightly ovular, with the outer margin with a row of pinnae similar to those along the posterior edge of the capsule, rarely forked, almost always with a single prominent end. Overall color light to medium brown (Figure 9E, F).

Measurements including the extended pinnae [mm]. Length (including operculum) 5.6–5.7, maximum width of capsule when viewed from lateral aspect 4.0–4.3 mm, length of micropylar plate 2.5–2.6 mm.

##### Newly hatched nymphs.

General color throughout the body (including head and antennae) is dark brown to black (Figure 12E). Margins of the abdomen are white. Meso- and metafemora with a continuous transverse white band on the proximal third; a small white spot on the interior lobe proximal margin; and a thin white crescent on the distal exterior lobe margin. Profemora dark brown to black, except for near the proximal third where there can be small white spots on each side of the profemoral shaft, but not a solid transverse white band. Tibiae on all legs the same dark brown to black as the rest of the body but with faint two to three tan spots on all of the interior margins on the proximal third, or more clearly white than tan on the protibial interior lobe. Basitarsi are white and remaining tarsal segments are tan to dark brown.

##### Etymology.

Patronym, named after Garda Bagus (Java, Indonesia) who has helped to collect and rear several *Phyllium* species over the years and who has been instrumental in getting these established in the phasmid breeding community.

### Checklist of species known from Sumatra and Java

The distribution is indicated by (S) for Sumatra; (J) for Java; or (S, J) for both islands.

Phyllium (Pulchriphyllium) giganteum Hausleithner, 1984 (S, J)

Phyllium (Pulchriphyllium) pulchrifolium Audinet-Serville, 1838 (S, J)

= *magdelainei* Lucas, 1857

Phyllium (Pulchriphyllium) bioculatum Gray, 1832 (S)

Phyllium (Pulchriphyllium) abdulfatahi Seow-Choen, 2017 (S)

Phyllium (Pulchriphyllium) shurei Cumming and Le Tirant, 2018 (J)

Phyllium (Phyllium) jacobsoni Rehn & Rehn, 1934 (J, Sumatran record presented in Seow-Choen (2017) is unconfirmed. Seow-Choen cites this record based on a single male specimen collected in 1936 from the NHMB collection. Unfortunately, due to old age and poor preservation of the specimen, the coxae color is faded removing the only easy and reliable currently known morphological method for differentiation. Hopefully further exploration on Sumatra can reveal if in fact *P.jacobsoni* is present or if this was simply a *P.nisus* sp. nov. male with the coxae color faded.)

Phyllium (Phyllium) nisus sp. nov. (S) (holotype Figure 13A)

Phyllium (Phyllium) gardabagusi sp. nov. (J) (holotype Figure 13B)

### Erroneous records

Phyllium (Phyllium) hausleithneri Brock, 1999. Only known from Peninsular Malaysia; the population from Sumatra discussed in Cumming et al. (2018) is now known as *Phylliumnisus* sp. nov., sister species to *Phylliumhausleithneri*.

Phyllium (Pulchriphyllium) mannani Seow-Choen, 2017. This species was erroneously recorded from Sumatra based on a male *Phylliumbioculatum* Gray, 1832 specimen from the NHMB collection and published in Seow-Choen (2018). Features which differentiate *P.bioculatum* from *P.mannani* are as follows:

1 Antennae length, *P.mannani* = 18–21 mm (shorter or same length as tegmina when at rest along the dorsal surface), *P.bioculatum* = 26–28 mm (antennae always longer than tegmina).

2 Tegmina length, *P.mannani* = 14–15.5 mm, *P.bioculatum* = 11–11.5 mm.

3 Widest abdominal segment, *P.mannani* = segment VI, *P.bioculatum* = segment V.

4 Protibial exterior lobe, *P.mannani* = nearly absent, only a slight sliver, *P.bioculatum* = distal end notably wider than the remainder of the length.

5 Mesofemoral exterior lobe shape, *P.mannani* = V-shaped, *P.bioculatum* = U-shaped.

To date no records of *Phylliummannani* from Sumatra are known to exist, and based on the above morphological characters, the specimen in the NHMB keys out as a *Phylliumbioculatum* male.

## Discussion

Our molecular results reveal that the two Javan species are the sister group of the Sumatran and Peninsular Malaysian sister species, *Phylliumnisus* and *Phylliumhausleithneri*, respectively. Although we were able to examine an extensive range of specimens for all three species with purple coxae, their wide range of morphological variation has not allowed reliable external morphological differentiation. There is however the possibility of internal genitalia structures which might allow morphological differentiation, but despite dissection of several individuals no such structures could be identified. With all purple coxae species morphologically inseparable as adults, only the eggs of *P.nisus* and *P.hausleithneri*, can be distinguishable by their coloration (dark brown in *P.hausleithneri* Figure 9A and tan in *P.nisus* sp. nov. Figure 9C). Sumatran *P.nisus* sp. nov. was considered a range expansion of *P.hausleithneri* from Peninsular Malaysia (Cumming et al. 2018) but both species are now inferred as separate species and sister taxa with good support (Figure 2). Our molecular results illustrate the value of molecular data compared to traditional morphological taxonomic work as based on our two newly described cryptic species, which would have remained undescribed otherwise.

The significance of wing venation traits for Phylliidae systematics has been emphasized before (Bradler 2009), and we found the fusion of the MA and MP towards the apex of the male hind wing and the subsequent fusion with Rs (Figure 10B) to be an apomorphic (derived) trait of the *Phyllium* clade B. In the females of this clade the tegmina exhibit a conspicuous short vein that connects Rs with M (approximately at one third of the wing length, Figure 10A, noted as R–M in our figure). This vein was mentioned and depicted by Klante (1976: figs 11, 12) for the erroneously identified *P.woodi* (see above) and might constitute another derived trait of taxonomic value for this clade. To corroborate these assumptions a thorough comparison of the wing venation across the whole Phylliidae is necessary, which is beyond the scope of the current contribution and will become the subject in more comprehensive future studies.

The purple coxae coloration observed in *P.hausleithneri* and the two newly described species *P.gardabagusi* and *P.nisus* is likely a homologous trait between these three taxa, given their close relationship as recovered here (Figure 2), which originated in the last common ancestor of clade B. The absence of purple coxae coloration in *P.jacobsoni*, which is nested within clade B, could be the result of secondary loss. Although the assumption of two independent origins of purple coxae is equally parsimonious as one gain and one loss, requiring two steps each, we favor a single origin of this unique trait. Considering the low node support, we cannot exclude a sister relationship of *P.jacobsoni* to *P.gardabagusi* + *P.hausleithneri* + *P.nisus* and, consequently, a single origin of purple coloration without secondary loss. Vibrant coxae colors are rare among leaf insects, such as orange for *Phylliumrubrum* Cumming et al., 2018 or black for *Phylliumgantungense* Hennemann et al., 2009 and obviously serve some unidentified purpose. Leaf insects, as most phasmatodeans, rely on camouflage with reduced diurnal activity to minimize the risk of detection. If their camouflage fails, phasmatodeans often have elaborate secondary defenses to deploy, for instance deimatic behavior that involves the sudden display of striking coloration in several species from the hind wings (Bedford 1978). In some taxa these vibrant colors associated with startle displays stem from membranes, for instance, the turquoise coloration between the sclerites on the venter of the metathorax and proximal abdomen of *Haaniellaechinata* (Hennemann et al. 2016). Suddenly revealing visual cues during deimatic behavior is thought to stop a predator’s attack or make it pause long enough for the insect to escape (Umbers et al. 2015). We tentatively speculate that Phylliidae also use their vibrantly colored coxal membrane for defense purpose, but we lack behavioral observations supporting this assumption.

## Outlook

The present study is a first step towards understanding the phylogeny, taxonomy, historical biogeography, and evolution of leaf insects. Preliminary molecular data indicated that the leaf insect genera *Phyllium* and *Chitoniscus* both do not form monophyletic groups (Buckley et al. 2009; Bradler et al. 2015; Robertson et al. 2018). Thus, the Phylliidae are in need of a thorough taxonomic revision based on robust phylogenetic background information. As discussed in our interpretation of the molecular analyses, some of the branches lack strong support values, therefore we cannot at this time present a fully reliable interpretation of their phylogenetic relationships within the two clades. Hopefully, with additional genetic data included and an enhanced taxon sampling in future works, we expect a more robust phylogeny to emerge.

## Supplementary Material

XML Treatment for Phyllium (Pulchriphyllium)

XML Treatment for Phyllium (Pulchriphyllium) giganteum

XML Treatment for Phyllium (Phyllium)

XML Treatment for Phyllium (Phyllium) jacobsoni

XML Treatment for Phyllium (Phyllium) hausleithneri

XML Treatment for Phyllium (Phyllium) nisus

XML Treatment for Phyllium (Phyllium) gardabagusi

## Supplementary materials

Supplementary material 1 Table S1. GenBank accession numbers for 55 sequences from *Phyllium* specimens, including sex, type designation, specimen collection code, and collection data https://binary.pensoft.net/file/381038This dataset is made available under the Open Database License (http://opendatacommons.org/licenses/odbl/1.0/). The Open Database License (ODbL) is a license agreement intended to allow users to freely share, modify, and use this Dataset while maintaining this same freedom for others, provided that the original source and author(s) are credited.Royce T. Cumming, Sarah Bank, Stephane Le Tirant, Sven Bradler

Supplementary material 2 Table S2. Primers and corresponding information for genes used in this study https://binary.pensoft.net/file/381039This dataset is made available under the Open Database License (http://opendatacommons.org/licenses/odbl/1.0/). The Open Database License (ODbL) is a license agreement intended to allow users to freely share, modify, and use this Dataset while maintaining this same freedom for others, provided that the original source and author(s) are credited.Royce T. Cumming, Sarah Bank, Stephane Le Tirant, Sven Bradler

Supplementary material 3 Table S3. Measurements of *Phylliumnisus* sp. nov., holotype female, para- type males and females https://binary.pensoft.net/file/381040This dataset is made available under the Open Database License (http://opendatacommons.org/licenses/odbl/1.0/). The Open Database License (ODbL) is a license agreement intended to allow users to freely share, modify, and use this Dataset while maintaining this same freedom for others, provided that the original source and author(s) are credited.Royce T. Cumming, Sarah Bank, Stephane Le Tirant, Sven Bradler

Supplementary material 4 Table S4. Measurements of *Phylliumgardabagusi* sp. nov., holotype female, paratype males and females Explanation note: All measurements made to the nearest 0.1 mm. Measurements for paratypes are given with a minimum to maximum range. *Including cerci and head, excluding antennae. **Only one female specimen [Coll RC 16-203] had the alae exposed to measure. https://binary.pensoft.net/file/381041This dataset is made available under the Open Database License (http://opendatacommons.org/licenses/odbl/1.0/). The Open Database License (ODbL) is a license agreement intended to allow users to freely share, modify, and use this Dataset while maintaining this same freedom for others, provided that the original source and author(s) are credited.Royce T. Cumming, Sarah Bank, Stephane Le Tirant, Sven Bradler

Supplementary material 5 File S1. Concatenated supermatrix of COI, 28S, and 16S sequences from the 21 specimens sampled (18 Phyllium and three outgroups) Explanation note: Supermatrix of 16 COI, 18 28S, and 21 16S sequences from the 21 specimens sampled (18 *Phyllium* and three outgroups). https://binary.pensoft.net/file/381042This dataset is made available under the Open Database License (http://opendatacommons.org/licenses/odbl/1.0/). The Open Database License (ODbL) is a license agreement intended to allow users to freely share, modify, and use this Dataset while maintaining this same freedom for others, provided that the original source and author(s) are credited.Royce T. Cumming, Sarah Bank, Stephane Le Tirant, Sven Bradler

Supplementary material 6 File S2. Original newick-formatted tree file inferred from COI, 28S, and 16S using IQ-TREE Explanation note: Original tree file based on 16 COI, 18 28S, and 21 16S sequences. https://binary.pensoft.net/file/381043This dataset is made available under the Open Database License (http://opendatacommons.org/licenses/odbl/1.0/). The Open Database License (ODbL) is a license agreement intended to allow users to freely share, modify, and use this Dataset while maintaining this same freedom for others, provided that the original source and author(s) are credited.Royce T. Cumming, Sarah Bank, Stephane Le Tirant, Sven Bradler

Supplementary material 7 File S3. Deposition of paratype material for Phyllium (Phyllium) gardabagusi sp. nov. and Phyllium (Phyllium) nisus sp. nov. https://binary.pensoft.net/file/381044This dataset is made available under the Open Database License (http://opendatacommons.org/licenses/odbl/1.0/). The Open Database License (ODbL) is a license agreement intended to allow users to freely share, modify, and use this Dataset while maintaining this same freedom for others, provided that the original source and author(s) are credited.Royce T. Cumming, Sarah Bank, Stephane Le Tirant, Sven Bradler
